# Sphingolipid Δ4-desaturation is an important metabolic step for glycosylceramide formation in *Physcomitrium patens*

**DOI:** 10.1093/jxb/erab238

**Published:** 2021-06-10

**Authors:** Jasmin Gömann, Cornelia Herrfurth, Krzysztof Zienkiewicz, Tegan M Haslam, Ivo Feussner

**Affiliations:** 1 Department of Plant Biochemistry, Albrecht-von-Haller Institute for Plant Sciences, University of Göttingen, Göttingen, Germany; 2 Service Unit for Metabolomics and Lipidomics, Göttingen Center for Molecular Biosciences (GZMB), University of Göttingen, Göttingen, Germany; 3 Department of Plant Biochemistry, Göttingen Center for Molecular Biosciences (GZMB), University of Göttingen, Göttingen, Germany; 4 Institute of Plant Molecular Biology, Academia Sinica, Taiwan

**Keywords:** Glycosylceramide, glycosylceramide synthase, long-chain base desaturation, non-vascular plants, *Physcomitrium patens*, plant development, sphingolipid Δ4-desaturase, sphingolipid metabolism

## Abstract

Glycosylceramides are abundant membrane components in vascular plants and are associated with cell differentiation, organogenesis, and protein secretion. Long-chain base (LCB) Δ4-desaturation is an important structural feature for metabolic channeling of sphingolipids into glycosylceramide formation in plants and fungi. In *Arabidopsis thaliana*, LCB Δ4-unsaturated glycosylceramides are restricted to pollen and floral tissue, indicating that LCB Δ4-desaturation has a less important overall physiological role in *A. thaliana*. In the bryophyte *Physcomitrium patens*, LCB Δ4-desaturation is a feature of the most abundant glycosylceramides of the gametophyte generation. Metabolic changes in the *P. patens* null mutants for the sphingolipid Δ4-desaturase (*PpSD4D*) and the glycosylceramide synthase (*PpGCS*), *sd4d-1* and *gcs-1*, were determined by ultra-performance liquid chromatography coupled with nanoelectrospray ionization and triple quadrupole tandem mass spectrometry analysis. *sd4d-1* plants lacked unsaturated LCBs and the most abundant glycosylceramides. *gcs-1* plants lacked all glycosylceramides and accumulated hydroxyceramides. While *sd4d-1* plants mostly resembled wild-type plants, *gcs-1* mutants were impaired in growth and development. These results indicate that LCB Δ4-desaturation is a prerequisite for the formation of the most abundant glycosylceramides in *P. patens*. However, loss of unsaturated LCBs does not affect plant viability, while blockage of glycosylceramide synthesis in *gcs-1* plants causes severe plant growth and development defects.

## Teaser

Desaturation of the long-chain base moiety of ceramide enables the formation of glycosylceramides in *Physcomitrium patens*. Complete blockage of glycosylceramide formation affects growth and normal development of protonema and gametophore tissues.

## Introduction

Sphingolipids are essential structural elements in eukaryotic membranes. They constitute up to 40 mol % of plant plasma membrane lipids (Tjellström *et al.*, 2010; Cacas *et al.*, 2016; Luttgeharm *et al.*, 2016). Together with sterols they form so called micro- and nanodomains that are thought to act as sorting platforms for membrane proteins (Borner *et al.*, 2005; Grosjean *et al.*, 2015, 2018). Sphingolipids can further act as second messengers during developmental processes and in response to biotic and abiotic stresses (Shi *et al.*, 2007; Alden *et al.*, 2011; Chen *et al.*, 2012; Guo *et al.*, 2012).

The involvement of sphingolipids in a multitude of plant physiological processes likely results from their great structural diversity. All sphingolipids have the same characteristic non-polar backbone with a long-chain base (LCB) as the key element. Plant LCBs typically have a chain length of 18 carbon atoms and may be *N*-acylated to a long-chain fatty acid or a very-long-chain fatty acid. LCBs that are connected to fatty acids are called ceramides. The chain length of the fatty acid moiety of plant sphingolipids ranges from 16 to 26 carbon atoms. Structural modifications in the LCB and in the fatty acid substantially increase the diversity of the sphingolipid pool.

The simpler sphingolipids, LCBs and ceramides, are low abundant molecules that constitute around 2% and 0.5%, respectively, of all sphingolipids in *Arabidopsis thaliana* leaves (Markham *et al.*, 2006; Markham and Jaworski, 2007). They are important messenger molecules in cellular functions including programmed cell death (Greenberg *et al.*, 2000; Liang *et al.*, 2003; Shi *et al.*, 2007; Alden *et al.*, 2011).

Most plant sphingolipids have a polar head group attached to their non-polar ceramide backbone. These sphingolipids are referred to as complex sphingolipids and are divided into glycosylceramides (GlcCers) and glycosyl inositolphosphorylceramides (GIPCs). GlcCers contain one sugar residue as head group. Plant GIPCs contain an inositol-1-phosphate with up to seven different sugar moieties (Buré *et al.*, 2011; Cacas *et al.*, 2013). GlcCers and GIPCs constitute around 34% and 64%, respectively, of all sphingolipids in *A. thaliana* leaves (Markham *et al.*, 2006). They have structural functions in plant membranes and their relative abundance and composition influence the plant’s response towards abiotic stresses such as drought and cold stress (Ng *et al.*, 2001; Coursol *et al.*, 2003; Nagano *et al.*, 2014). The composition of complex sphingolipids likely affects trafficking of secretory proteins and signal transduction through the formation of microdomains (Simons and Toomre, 2000; Melser *et al.*, 2010).

Whether sphingolipids are channelled into GlcCer or GIPC formation is likely determined by certain LCB modifications. After their formation, LCBs harbour two hydroxyl groups at the C-1 and C-3 positions. The initial LCBs are thus referred to as dihydroxy LCBs, or, in short, d18:0 when palmitoyl-CoA is the acyl-CoA substrate. A third hydroxyl group may be introduced at the C-4 position through the action of an LCB C-4 hydroxylase, and the resulting LCBs are referred to as trihydroxy LCBs, or, in short, t18:0 (Sperling *et al.*, 2001; Chen *et al.*, 2008; Gömann *et al.*, 2021). Dihydroxy LCBs are enriched in GlcCers, while trihydroxy LCBs are enriched in GlcCers and GIPCs (Markham *et al.*, 2006).

Another crucial LCB modification is the insertion of double bonds. Double bonds may be introduced at the Δ4 or the Δ8 position by the activity of distinct LCB desaturases (Napier *et al.*, 2002). LCB C-4 hydroxylation and LCB Δ4-desaturation both act on the C-4 position of d18:0 LCBs and are therefore mutually exclusive. The resulting LCBs are referred to as d18:1 or d18:2 and t18:1, depending on the hydroxylation state and the number of inserted double bonds. Sphingolipids with Δ8-unsaturated LCBs predominate in all *A. thaliana* sphingolipid classes and in most tissues (Markham *et al.*, 2006). LCB Δ4-desaturation, however, mostly occurs in combination with LCB Δ8-desaturation and is restricted to GlcCers of *A. thaliana* floral and pollen tissue (Michaelson *et al.*, 2009).

The LCB Δ4-desaturases are a class of desaturases that is evolutionarily distinct from LCB Δ8-desaturases (Napier *et al.*, 2002). Knockout of the *A. thaliana* LCB Δ4-desaturase caused a significant reduction of pollen GlcCers compared with the wild type. However, its loss had no effect on plant morphology and physiology (Michaelson *et al.*, 2009). LCB Δ4-desaturation is therefore considered a less important LCB modification during *A. thaliana* sphingolipid biosynthesis. However, the LCB Δ4-desaturase appears to play a more important role in other plant species outside the Brassicaceae family. In tomato (*Solanum lycopersicum*) and soybean (*Glycine max*), Δ4,8-diunsaturated LCBs are enriched in GlcCers throughout the plant (Sperling *et al.*, 2005; Markham *et al.*, 2006).

The occurrence of LCB Δ4-unsaturated sphingolipids exemplifies how sphingolipid composition is tissue and organism dependent (Sperling *et al.*, 2005; Luttgeharm *et al.*, 2016). This highlights the importance of using appropriate model systems to study individual molecular species and classes. While *A. thaliana* has been an invaluable model for studying plant sphingolipid metabolism in general, it has limitations in the study of LCB Δ4-desaturation.

To better understand the physiological role of LCB Δ4-desaturation, the research focus should be directed towards plants outside the Brassicaceae family. Islam *et al.* (2012) analysed LCBs from 21 plant species from different phylogenetic groups to determine and compare the position of the double bond in d18:1. Among the 21 surveyed plants was the bryophyte *Physcomitrium* (formerly *Physcomitrella*) *patens*. The d18:1 LCB of *P. patens* has the double bond at the Δ4 position, and *P. patens* therefore qualifies as a suitable plant system to study the role of Δ4-unsaturated sphingolipids in plants.

**Fig. 1.**
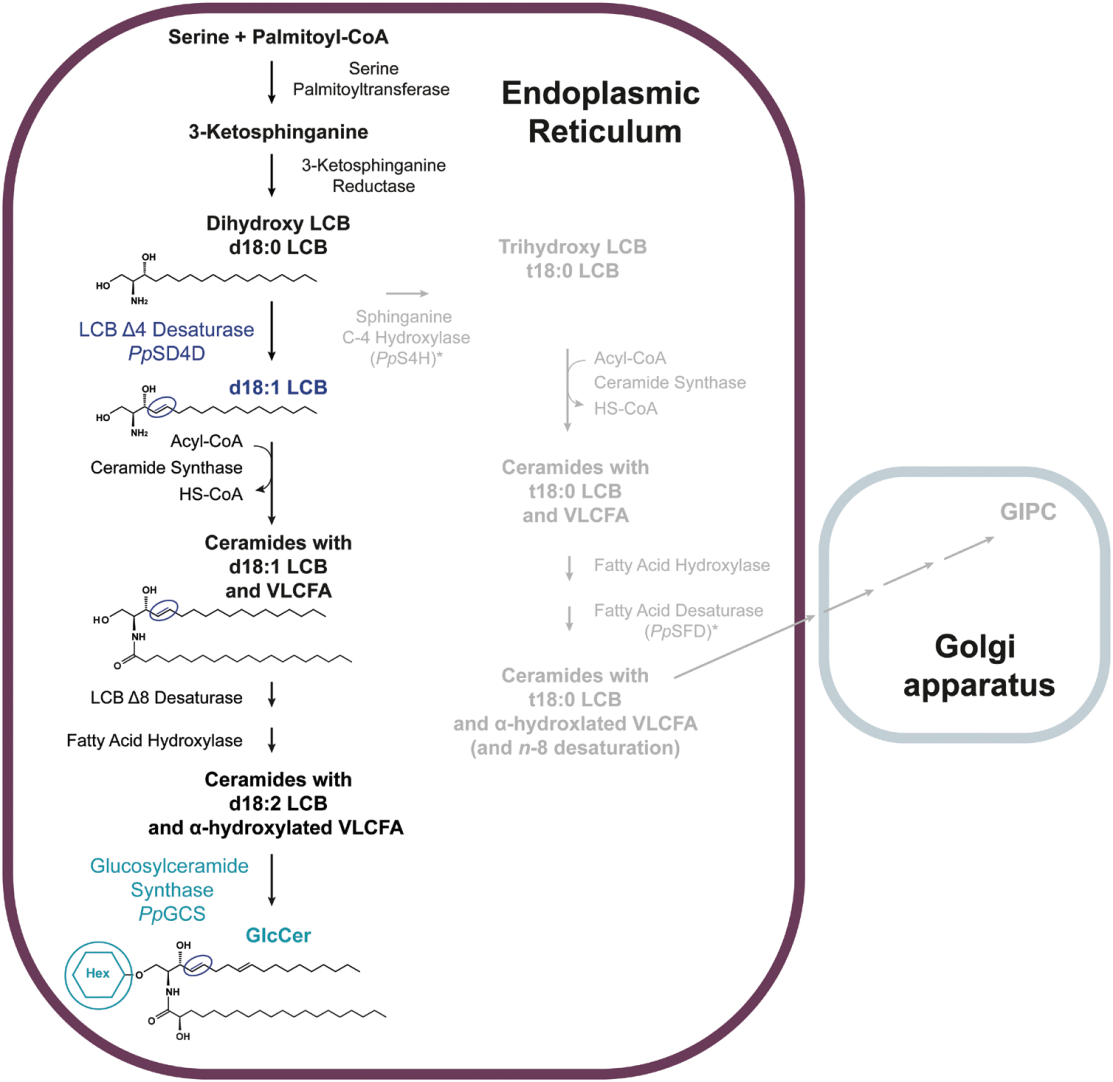
Sphingolipid biosynthesis in *P. patens*. This proposed schematic for sphingolipid metabolism in *P. patens* depicts downstream reactions for glycosylceramide (GlcCer) and glycosyl inositolphosphorylceramide (GIPC) synthesis. Dihydroxy long-chain bases (LCBs) with ∆4 and ∆8 double bonds are mainly designated for GlcCer formation (coloured), while trihydroxy LCBs are mainly designated for GIPC formation (grey). Asterisks indicate functionally characterized enzymes in *P. patens*. Hex, hexose; HS-CoA, Coenzyme A; SFD, sphingolipid fatty acid desaturase; VLCFA, very-long-chain fatty acid; UDP-Glc, uridine diphosphate glucose.

A recent study described the lipidome of *P. patens*, including the four sphingolipid classes: LCBs, ceramides, GlcCers, and GIPCs (Resemann *et al.*, 2021). In *P. patens* there is a clear distinction between specific LCB modifications that occur in GlcCers and those that occur in GIPCs. Over 90% of GlcCers have a d18:2 LCB in their backbone, whereas GIPCs have only a t18:0 LCB. This structural distinction facilitates study of the role of different sphingolipid species in plant physiology. The data further suggest that the physiological role of Δ4-unsaturated sphingolipids is tightly bound to the role of GlcCers in *P. patens*. A proposed schematic for the metabolic flux of sphingolipid precursors in *P. patens* was generated based on sphingolipid data from Resemann *et al.* (2021) and information about sphingolipid biosynthesis in *A. thaliana* (Luttgeharm *et al.*, 2016, and is shown in Fig. 1.

Although GlcCers are the second most abundant sphingolipids found in *A. thaliana*, their physiological function is not fully understood. GlcCers are generated by the transfer of a glucose residue from uridine diphosphate glucose to the ceramide backbone. This reaction is catalysed by a glycosylceramide synthase (GCS). *Arabidopsis thaliana* has one *GCS* gene (At2g19880) (Msanne *et al.*, 2015); *gcs* null mutants lacked all GlcCers and had a higher GIPC content than wild-type plants. The mutants were seedling lethal and were impaired in cell type differentiation and organogenesis (Msanne *et al.*, 2015).

In this study, the physiological roles of sphingolipids with Δ4-unsaturated LCBs and of GlcCers were investigated in *P. patens*. The LCB Δ4-desaturase and the GCS, designated here as PpSD4D and PpGCS, respectively, were identified in *P. patens* by sequence similarity to their *A. thaliana* orthologs. Gene knockouts revealed an inhibition of GlcCer formation in both *sd4d* and *gcs* mutant lines. Although GlcCers were nearly absent in both mutants, *sd4d-1* and *gcs-1* plants had different phenotypes: *sd4d-1* plants had almost no morphological impairments, whereas *gcs-1* plants had substantial growth and development defects. Overall, the results indicate substantial flexibility with respect to the amount of GlcCer essential for plant survival, as well as developmental consequences of changes in GlcCer and ceramide precursor content.

## Materials and methods

### Plant material and growth conditions

The *P. patens* ‘Gransden’ strain (Hedw.) Bruch & Schimp was used as the wild type. Plants were grown under a 16 h light/8 h dark cycle at 25 °C and with a light intensity of 50–70 µmol m^−2^ s^−1^. Protonema was cultivated on BCD agar medium plates (90 mm diameter) containing 1 mM CaCl_2_ and 5 mM ammonium tartrate (BCDAT) (Ashton and Cove, 1977). For protonema cultivation the medium plates were covered with sterile cellophane discs (folia, Wendelstein, Germany). For protonema maintenance and propagation, 1- to 2-week-old tissue was scraped off the cellophane and disrupted in sterile water for 20 s using a tissue lyser (Ultra Turrax, Ika, Staufen, Germany). The cell suspension was spread on to fresh BCDAT plates. Plates were sealed with micropore tape before incubation.

Protonema material for lipid analysis was cultivated on cellophane-covered BCD plates. To compare measurements of different mutant lines, the dry weight of the disrupted material was determined after treatment with the tissue lyser. All plate cultures were subsequently started with a volume of cell suspension equal to 5 mg dry weight. To obtain enough material for lipid measurements, protonema from eight plates was pooled. The protonema was cultivated for 10 days before harvesting and was immediately frozen in liquid nitrogen after harvest. Prior to lipid extraction, the plant material was lyophilized. Plant growth was quantified by determination of the fresh weight of protonema after harvesting and before freezing in liquid nitrogen. Protonema was weighed again after lyophilization for determination of the dry weight. This experiment was part of a larger experiment and the lipid data for wild type protonema were used recently in a different study as well (Gömann *et al.*, 2021).

For imaging of gametophore development, 1 mm spot inocula of 7- to 10-day-old protonema tissue were placed on plates containing BCD medium with 1 mM CaCl_2_. Fully grown gametophores were imaged after 6 weeks. For protonema development, colonies were imaged after 1–2 weeks. Plates were sealed with micropore tape during cultivation.

Dark growth experiments for skotonema induction were performed as described in Saavedra *et al.* (2015). For these experiments, protonema spot inocula were placed on square BCDAT plates supplemented with 2% sucrose. Colonies were grown for 1 week under continuous light and subsequently moved to the dark and rotated into vertical orientation. Colonies were grown for another 4 weeks before imaging.

Images were captured using a binocular (Olympus SZX12 binocular, Olympus Corporation, Tokyo, Japan) or a microscope (Olympus BX51 microscope, Olympus Corporation, Tokyo, Japan) connected to a camera (R6 Retiga camera, QImaging, Surrey, Canada). Images were captured with the Ocular scientific image acquisition software (version 1.0, Digital Optics Ltd, Auckland, New Zealand). Images were processed using ImageJ 1.52b software (Schneider *et al.*, 2012).

### Generation of targeted knockout plasmids

Targeted knockout plasmids were assembled by cloning 750 bp fragments from the 5′ and the 3′ genomic DNA untranslated regions (UTR) of the respective *SD4D* and *GCS* genes into a pBluescript vector backbone. The 5′ and 3′ fragments flanked a kanamycin resistance cassette for future selection of knockout mutants. The following primer pairs [forward (fw) and reverse (rev)] were used for cloning the flanking regions of *SD4D* and *GCS*: 5′ *Sac*I *SD4D*-fw (5′-gagctcATGGACTTCTACTGGGCTGAGG-3′)/5′ *BamH*I *SD4D*-rev (5′-ggatccTCCTGACTCTAAGAAAGAAAAGTATAG-3′) and 3′ *Hind*III *SD4D*-fw (5′-gtcgacCTTCTATGCGTTCAGGCCTCTC-3′)/3′ *Apa*I *SD4D*-rev (5′-gggcccTCAGTTGGTTTTGCCATGCTTTGTC-3′). The following primer pairs were used for *GCS* cloning: 5′ *Sac*I *GCS*-fw (5′-gagctcATGGCGTTTGTGGAGGCCATG-3′)/5′ *Xba*I *GCS*-rev (5′-tctagaCCAATACCTGACTACGCCAATTGC-3′) and 3′ *Hind*III *GCS*-fw (5′-aagcttGTGATTTTTGTGAACTCAGTGAAATTG-3′)/3′ *Apa*I *GCS*-rev (5′-gggcccTCATTGTACCTGACAAATGTTTCCATT-3′). Correct cloning of the fragments into the destination vector was confirmed by plasmid sequencing. To linearize the *SD4D* and *GCS* fragments used for *P. patens* homologous recombination, the restriction enzymes *Sac*I and *Apa*I were used for both gene constructs.

### Transformation of *P. patens* and molecular characterization of knockout mutants

Knockout plants were generated via polyethylene glycol-mediated transformation of *P. patens* protoplasts according to Schaefer *et al.* (1991). The knockout constructs containing the 5′ and 3′ *SD4D* and *GCS* flanking regions and the kanamycin selection cassette were used for homologous recombination in *P. patens*. To confirm the correct insertion of the selection cassette into the *P. patens* genome, genomic DNA was extracted from a small sample of gametophytic tissue. DNA was isolated using cetyl trimethylammonium bromide extraction. In a first step, successful integration of the kanamycin selection cassette was confirmed by PCR using the following primer combination: kan fw: 5′-ATGGGGATTGAACAAGATGGATTGCAC-3′/kan rev: 5′-TCAGAAGAACTCGTCAAGAAGGC-3′. In a second PCR, insertion of the selection cassette into the *SD4D* and *GCS* sites was confirmed. The following primer combinations were used to confirm insertion into the *SD4D* locus: 5′ UTR (fw: 5′-GTGGTGTGGTTGCCGTCAAGAC-3′/rev: 5′-TAGGGTTCCTATAGGGTTTCGCTC-3′), 3′ UTR (fw: 5′-GATAGCTGGGCAATGGAATCCG-3′/rev: 5′-GCATATTGTGGGTGCTGATGATTAGG-3′). The following primer combinations were used to confirm insertion into the *GCS* locus: 5′ UTR (fw: 5′-GCAACAATGTGCCCGAGCAGATC-3′/rev: 5′-TAGGGTTCCT ATAGGGTTTCGCTC-3′), 3′ UTR (fw: 5′-GATAGCTGGGCAA TGGAATCCG-3′/rev: 5′-GATGCAGATGATAAGGAGAA TCTCAGC-3′).

### Reverse transcription PCR verification of mutants

RNA was obtained from *P. patens* wild-type and mutant gametophytic tissue using TRIzol^TM^ reagent according to the manufacturer’s instructions (Thermo Fisher Scientific, Waltham, MA, USA). Total RNA was treated with DNAseI (Thermo Fisher Scientific, Waltham, MA, USA) prior to cDNA synthesis. A 1 µg aliquot of the DNAseI-treated RNA was used for cDNA synthesis. cDNA was synthesized using the RevertAid H Minus First Strand cDNA Synthesis Kit (Thermo Fisher Scientific, Waltham, MA, USA). The following primer pairs were used for amplification of fragments of *SD4D* (fw: 5′-CCGGTTGCTTGGCATATTCG-3′/rev: 5′-CAATGGGGTGCATACCACCT-3′), *GCS* (fw: 5′-CGCGTTATCAGCTCACCAGA-3′/rev: 5′-TCCTTCCC AGGTGACAATGC-3′), and *ACTIN8* transcript (fw: 5′-GCTGGTTT CGCTGGAGACGATGC-3′/rev: 5′-ATCGTGATCACCTGCCC GTCC-3′).

### Gene editing by CRISPR-Cas9

CRISPR-Cas9 was performed using the plasmid pUC57-PpU6pro-2XBbsI-etracRNA generated in-house. Gene expression was driven by the U6 promoter used in Collonnier *et al.* (2017). The inserted etracRNA was described in Castel *et al.* (2019). A dual *Bbs*I site was included in the plasmid for cloning. Prediction of protospacers was done using the CRISPOR software (Haeussler *et al.*, 2016) (http://tefor.net/crispor/crispor.cgi). The sgRNA gcs-1 was designed to target the first exon of *PpGCS*. Oligos for gcs-1 sgRNA: gcs-1 Oligo 1 (5′-AACCGGGATGGCAGAACACTAAGC-3′)/gcs-1 Oligo 2 (5′-AAACGCTTAGTGTTCTGCCATCCC-3′) were aligned and cloned into the pUC57-PpU6pro-2XBbsI-etracRNA vector. Successful cloning was confirmed by sequencing. The plasmid carrying the sgRNA was co-transformed with pAct-Cas9 (Collonnier *et al.*, 2017) and pBNRF (Schaefer *et al.*, 2010), which carries resistance to G418 (Geneticin), into *P. patens* protoplasts. pAct-Cas9 and pBNRF were both kindly provided by Prof. Fabien Nogué, INRA Versailles-Grignon, France. Polyethylene glycol-mediated protoplast transformation was performed as described before. Putative knockout mutants were confirmed by sequencing of the PCR product using the following primer combination that surrounded the target sequence: gcs-1-fw (5′-GGAGATGCGGTGAGAAGAAAC-3′)/gcs-1-rev (5′-TAAACC CCCACGATCACTGC-3′).

### Sphingolipid extraction and analysis

Sphingolipid extraction was achieved by application of the lipid extraction protocol described in Markham *et al.* (2006) with minor modifications. Lipids were extracted from 20 mg of lyophilized and homogenized *P. patens* protonema material. The tissue was extracted at 60 °C using an extraction solvent composed of propan-2-ol/hexane/water (60:26:14, v/v/v). Lipids were resuspended in 800 µl of a final solvent mixture composed of tetrahydrofuran/methanol/water (4:4:1, v/v/v). Samples were chemically modified or directly analysed with ultra-performance liquid chromatography (UPLC) coupled with nanoelectrospray ionization (nanoESI) and triple quadrupole tandem mass spectrometry (MS/MS) (AB Sciex, Framingham, MA, USA) for measurement of LCBs.

### Methylamine treatment

Lipid extracts were treated with methylamine solution for the analysis of ceramides, GlcCers, and GIPCs. A 50 µl volume of the lipid extract was evaporated. Dried lipids were resuspended in 1.4 ml of 33% (w/v) methylamine dissolved in ethanol and 600 µl of water (Markham and Jaworski, 2007). The methylamine/lipid mixture was incubated for 1 h at 50 °C. The solvent was subsequently evaporated, and the dried lipids were dissolved in 50 µl tetrahydrofuran/methanol/water (4:4:1, v/v/v). The samples were used for UPLC-nanoESI-MS/MS analysis.

### Derivatization with acetic anhydride

LCB phosphates (LCB-Ps) were detected after acetic anhydride derivatization using a modified protocol from Berdyshev *et al.* (2005) and Yanagawa *et al.* (2017). A 50 µl volume of the lipid extract was evaporated and the dried lipids were dissolved in 100 µl pyridine and 50 µl acetic anhydride. Derivatization was performed at 50 °C for 30 min. The solvent mixture was subsequently evaporated, and samples were dissolved in 50 µl tetrahydrofuran/methanol/water (4:4:1, v/v/v). The samples were used for UPLC-nanoESI-MS/MS analysis.

### Lipid analysis

Measurement of the molecular lipid species was performed using the UPLC-nanoESI-MS/MS with multiple reaction monitoring approach described in Resemann *et al.* (2021) and Herrfurth *et al.* (2021). All multiple reaction monitorings for the distinct sphingolipid classes were measured for the putative lipid species having d18:0, d18:1, d18:2, t18:0, and t18:1 as LCB residues and chain lengths from C16 to C28 as acyl residues that are saturated or monounsaturated and unhydroxylated or monohydroxylated (Supplementary Table S1). LCB-Ps were measured in negative ionization mode with [M-H]^−^ as precursor ions. Series A and series B GIPC classes were analysed in positive ionization mode with [M+NH_4_]^+^ as precursor ions and ceramides as fragment ions. Determination of head-group-specific ions was done as described before (Buré *et al.*, 2011). UPLC-nanoESI-MS/MS data were processed using Analyst 1.6.2 and MultiQuant 3.0.2 software (both AB Sciex, Framingham, MA, USA).

### Web tools

#### BLAST search

*Physcomitrium patens* sphingolipid Δ4-desaturase and GCS were identified via sequence homology to the corresponding *A. thaliana* proteins. A BLAST search for *P. patens* was performed using the National Center for Biotechnology Information (NCBI) proteome database (National Library of Medicine, Bethesda, MD, USA; http://www.ncbi.nlm.nih.gov/BLAST/) (Altschul *et al.*, 1990).

#### Transmembrane domain prediction

Transmembrane domain prediction for PpSD4D and PpGCS was done using the TMHMM software (Sonnhammer *et al.*, 1998; Krogh *et al.*, 2001).

#### Gene expression

Information about *PpSD4D* and *PpGCS* gene expression was obtained using the *P. patens* electronic fluorescent pictograph (eFP) browser (http://www.bar.utoronto.ca) (Winter *et al.*, 2007; Ortiz-Ramírez *et al.*, 2016).

## Results

### Protein sequence similarity indicates that *PpSD4D* and *PpGCS* are single genes with similar expression patterns in *P. patens*

Candidate orthologs of the characterized *A. thaliana* sphingolipid Δ4-desaturase (At4g04930) (Michaelson *et al.*, 2009) and GCS (At2g19880) (Msanne *et al.*, 2015) were identified in the *P. patens* proteome via a BLAST search of the NCBI database. PpSD4D (XP_024361943.1) and PpGCS (XP_024399720.1) had 63% and 57% identity, respectively, to their *A. thaliana* orthologs. Based on sequence similarity, both *P. patens* proteins are considered to be encoded by single genes. PpSD4D is an enzyme of 382 amino acids. Like its *A. thaliana* counterpart, PpSD4D does not have an N-terminal cytochrome b5 fusion domain (Napier *et al.*, 1999; Sperling *et al.*, 2003). PpSD4D includes three histidine boxes that are characteristic for membrane-bound desaturases and hydroxylases and that coordinate the di-iron cluster in the active site (Shanklin and Cahoon, 1998; Bai *et al.*, 2015). PpGCS is an enzyme of 518 amino acids with a conserved glycosyl transferase domain.

Using TMHMM software, transmembrane domains were predicted for both proteins (Supplementary Fig. S1A, B). According to these predictions, PpSD4D contained four transmembrane domains and PpGCS contained two transmembrane domains. The expression patterns reported in the eFP browser (Winter *et al.*, 2007; Ortiz-Ramírez *et al.*, 2016) revealed that *PpSD4D* and *PpGCS* had similar expression in *P. patens* tissues (Supplementary Fig. S1C, D). The highest expression was in the protonema, the spores, and the sporophyte generation. *PpGCS* had a generally higher expression than *PpSD4D*. The similar expression patterns would be consistent with the notion that both genes could contribute to the same processes. This idea is supported by the *P. patens* sphingolipid profile identified recently, in which LCB Δ4-desaturation seems to be a prerequisite for GlcCer formation (Resemann *et al.*, 2021).

### GlcCers are nearly absent in *sd4d* and *gcs* plants

**Fig. 2.**
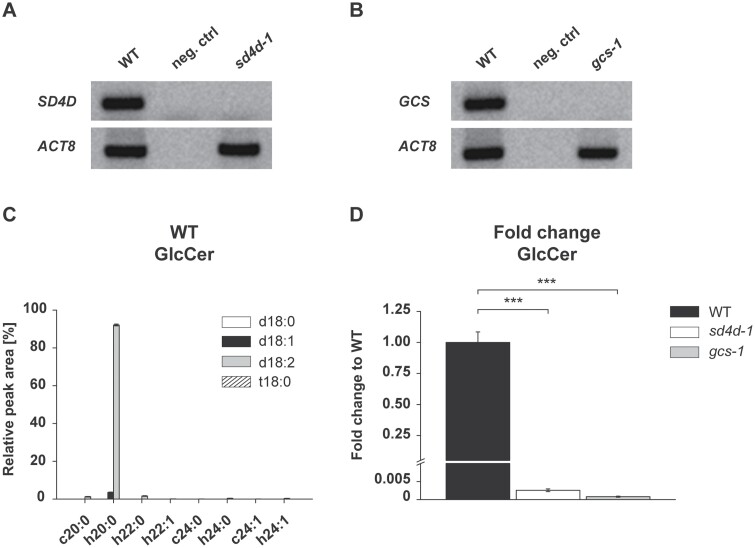
Characterization of *P. patens sd4d-1* and *gcs-1* mutants and analysis of the GlcCer content of *P. patens* wild type (WT), *sd4d-1*, and *gcs-1*. (A) *PpSD4D* and (B) *PpGCS* transcript determination by reverse transcription PCR. *ACTIN8* (*ACT8*) was used as the reference gene and water as the negative control (neg. ctrl). (C, D) GlcCers were extracted from protonema of 10-day-old WT, *sd4d-1*, and *gcs-1 P. patens* and analysed with UPLC-nanoESI-MS/MS. (C) Relative GlcCer profile of *P. patens* WT. GlcCers are shown with their LCB (column shading) and fatty acid (x-axis) moieties. Dihydroxy LCBs are indicated by a ‘d’ and trihydroxy LCBs are indicated by a ‘t’. Molecular species with unhydroxylated fatty acids are indicated by a ‘c’ and molecular species with α-hydroxylated fatty acids are indicated by an ‘h’. Only molecular species with a peak area ≥0.1% are included in the GlcCer graphs. Please note that this experiment was part of a larger experiment and the data for wild type protonema were shown recently in a different study as well (Gömann *et al.*, 2021). (D) GlcCer fold changes relative to the WT were calculated using absolute peak areas. Fold changes are depicted on a linear scale. The WT is set to a value of 1. Sphingolipid data represent the mean ±SD of measurements from four independent cultivations, each containing protonema material from eight cultivation plates. Statistical analysis was done using a two-tailed Student’s *t*-test. Asterisks indicate significant differences from the WT: ***P<0.001.

To determine the enzymatic function of PpSD4D and PpGCS *in planta*, knockout mutants for both single genes were generated by homologous recombination. Seven independent *sd4d* knockouts were obtained. The absence of the *PpSD4D* transcript in these mutants was confirmed by semi-quantitative reverse transcription PCR (Fig. 2A, Supplementary Fig. S2A). In contrast, only a single true *gcs* mutant was obtained by homologous recombination (Fig. 2B, Supplementary Fig. S2B). CRISPR-Cas9 genome editing was therefore additionally applied to target *PpGCS*. Three more mutants were generated using CRISPR-Cas9 targeting the first exon (Supplementary Fig. S2C). These mutants showed the same growth phenotype as the *gcs-1* mutant obtained by homologous recombination. The knockout was confirmed by sequencing of the targeted gene region (Supplementary Fig. S2D). All three mutants had frame-shift deletions that interfered with proper translation of the protein.

**Fig. 3.**
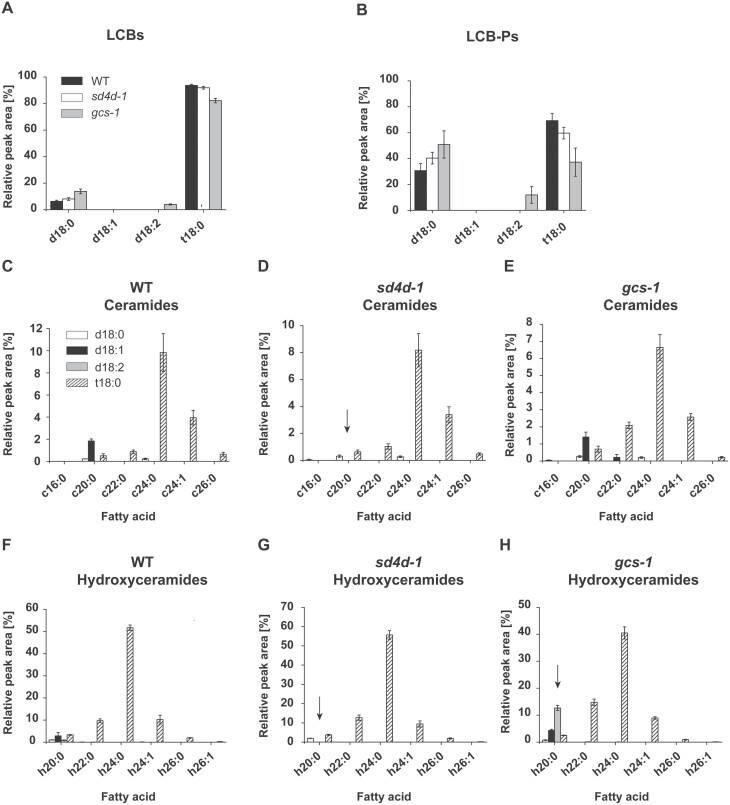
Relative profiles of LCBs, LCB-Ps, and ceramides in *P. patens* wild type (WT), *sd4d-1*, and *gcs-1*. LCBs, LCB-Ps, and ceramides were extracted from protonema of 10-day-old WT, *sd4d-1*, and *gcs-1 P. patens* and analysed with UPLC-nanoESI-MS/MS. Relative profiles of (A) LCBs, (B) LCB-Ps, and (C–H) ceramides in WT, *sd4d-1*, and *gcs-1* lines are shown. Dihydroxy LCBs are indicated by a ‘d’ and trihydroxy LCBs are indicated by a ‘t’. Molecular species with unhydroxylated fatty acids are indicated by a ‘c’ and molecular species with α-hydroxylated fatty acids are indicated by an ‘h’. Please note that this experiment was part of a larger experiment and the data for wild type protonema were shown recently in a different study as well (Gömann *et al.*, 2021). Relative profiles of (C–E) ceramide and (F–H) hydroxyceramide molecular species are shown with their LCB (column shading) and fatty acid (x-axis) moieties. Arrows highlight changes relative to the WT. Sphingolipid data represent the mean ±SD of measurements from four independent cultivations, each containing protonema material from eight cultivation plates.

**Fig. 4.**
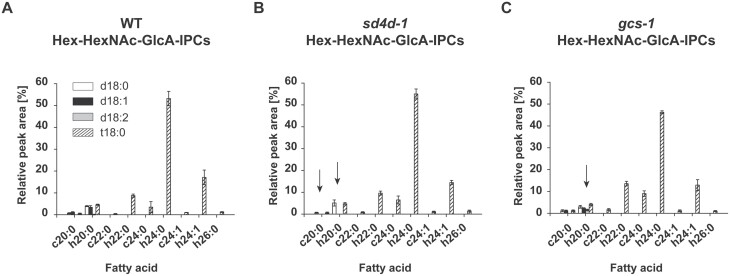
Relative Hex-HexNAc-GlcA-IPC profiles in *P. patens* (A) wild type (WT), (B) *sd4d-1*, and (C) *gcs-1*. GIPCs with one Hex moiety and one HexNAc unit (Hex-HexNAc-GlcA-IPCs) were extracted from protonema of 10-day-old WT, *sd4d-1*, and *gcs-1 P. patens* and analysed with UPLC-nanoESI-MS/MS. Only molecular species with a peak area ≥0.5% in at least one of the three lines are included in the graphs. Hex-HexNAc-GlcA-IPC molecular species are shown with their LCB (column shading) and fatty acid (x-axis) moieties. Dihydroxy LCBs are indicated by a ‘d’ and trihydroxy LCBs are indicated by a ‘t’. Molecular species with unhydroxylated fatty acids are indicated by a ‘c’ and molecular species with α-hydroxylated fatty acids are indicated by an ‘h’. Arrows highlight changes relative to the WT. Sphingolipid data represent the mean ±SD of measurements from four independent cultivations, each containing protonema material from eight cultivation plates.

Lipid measurements were conducted on *P. patens* wild-type and the *sd4d* and *gcs* mutants to determine their sphingolipid composition. For better visualization of the ceramide backbone composition, molecular species of GlcCers (Fig. 2C), ceramides (Fig. 3C–H), and GIPCs (Fig. 4) were divided into species with unhydroxylated fatty acids (indicated by a ‘c’ before the chain length number) and species with α-hydroxylated fatty acids (indicated by an ‘h’ before the chain length number). Lipids were extracted from 10-day-old protonema grown on cellophane-covered BCD medium and analysed by UPLC-nanoESI-MS/MS. Growth on cellophane-covered medium enabled easy harvesting of the filamentous tissue. Previous analyses of *P. patens* sphingolipids showed that ~94% of GlcCers contain the Δ4,8-diunsaturated LCB moiety with two hydroxyl groups, d18:2 (Resemann *et al.*, 2021).

The *P. patens* wild-type GlcCer profile described recently by Resemann *et al.* (2021) was confirmed in this study (Fig. 2C). The profile suggested that GlcCer formation might be disturbed in the *sd4d* and *gcs* mutants. The lipid data therefore served as a second line of evidence for the functional disruption of the *PpSD4D* and *PpGCS* genes. Fold changes in GlcCer content compared with the wild type were determined to show the abolishment of GlcCers in the mutant lines. All tested *sd4d* and *gcs* lines had substantially reduced GlcCer levels compared with the wild type (Fig. 2D, Supplementary Fig S3). After confirmation of several independent knockout lines for each gene, all subsequent mutant characterizations were performed on the *sd4d-1* and *gcs-1* lines. In *sd4d-1* and *gcs-1*, GlcCer levels were reduced by 99.8% and 99.9%, respectively (Fig. 2D). More than 92% of the wild-type GlcCer pool consisted of a single sphingolipid species (Resemann *et al.*, 2021). This had a d18:2 LCB that was conjugated to an α-hydroxylated 20-carbon fatty acid with no double bonds, h20:0, together d18:2/h20:0. Minor species such as d18:1/h20:0, d18:2/h22:0, and d18:2/c20:0 accounted for 4%, 2%, and 1% of *P. patens* GlcCers, respectively. All other detected species represented <1% of total GlcCers. Interestingly, the *sd4d-1* mutant still had residual amounts of GlcCer d18:2/h20:0 that might derive from a putative desaturase activity of the LCB C-4 hydroxylase (Ternes *et al.*, 2002). *sd4d-1* plants also contained GlcCer species with a d18:0 LCB. However, all GlcCer species in *sd4d-1* were present in trace amounts and therefore did not produce a substantial GlcCer pool (Supplementary Tables S2–S4). The residual amounts of GlcCers found in the *gcs-1* mutant were attributed to background signals that were used to calculate the overall fold change. The GlcCer results verified the generation of null mutants for both genes and confirmed that PpSD4D and PpGCS are the only enzymes in *P. patens* that catalyse the respective reactions in the tested conditions and tissues.

### Loss of GlcCers and of Δ4-unsaturated LCBs affects the relative profiles of other sphingolipid classes

Sphingolipidomics also revealed changes in the profiles of other sphingolipid classes upon loss of either PpSD4D or PpGCS activity. The most abundant LCB and LCB-P species in the wild type was t18:0, constituting 94% and 69%, respectively (Fig. 3A, B). Lesser amounts of d18:0 were also found in LCB (6%) and LCB-P (31%). The overall LCB and LCB-P profiles were maintained in the *sd4d-1* and *gcs-1* mutants. However, minor changes were observed in the relative abundances of individual species. In the *sd4d-1* mutant, t18:0 LCB was reduced to 91%, and LCB-P to 60%. *sd4d-1* plants further had slight increases of d18:0 LCB to 8% and d18:0 LCB-P to 40%. The *gcs-1* mutant had lower t18:0 LCB, at 82%, and LCB-P, at 37%. *gcs-1* plants also had more d18:0 LCB, at 14%, and LCB-P, at 51%, compared with the wild type. Additionally, d18:2, which was not found in the wild type, emerged as a new LCB, at 4% of the total LCB content, and LCB-P, at 12% of the total LCB-P content, in *gcs-1*.

In the wild type, ceramides harbouring the t18:0 LCB predominated, constituting more than 90% of all ceramides (Fig. 3C, F). Only minor amounts of ceramides with d18:0, d18:1, and d18:2 LCBs were detected. The most abundant fatty acid, at 52% of the total, was h24:0, followed by fatty acids with carbon chain lengths ranging from C20 to C26. *sd4d-1* plants had similar ceramide profiles to the wild type (Fig. 3D, G). However, no ceramide species with d18:1 and d18:2 LCBs were found. The *gcs-1* mutant also had comparable ceramide profiles to the wild-type control (Fig. 3E, H). *gcs-1* specifically accumulated the d18:2/h20:0 ceramide species. Taken together, the data showed that *sd4d-1* lacked ceramides with d18:1 and d18:2 LCBs. *gcs-1* plants accumulated d18:2 LCBs and LCB-Ps, as well as the ceramide that is the characteristic backbone of the most abundant wild-type GlcCer species, d18:2/h20:0 (Fig. 2C), indicating that this might be the main substrate of PpGCS. However, this accumulation is minor relative to that of the t18:0 ceramides that predominate in *P. patens* (Fig. 3). It is likely that there is robust regulation of the accumulation of different ceramide species, perhaps mitigated by ceramidases, ceramide kinases, or other as yet unidentified enzymes.

GlcCers and GIPCs both contain polar head groups at the C-1 position of the LCB. Depending on the LCB hydroxylation and desaturation state of ceramides, either GlcCers or GIPCs are synthesized. GlcCer and GIPC formation represent alternative sphingolipid metabolic pathways and, therefore, the blockage of GlcCer synthesis in the *sd4d-1* and *gcs-1* mutants might result in changes in the synthesis and composition of GIPCs. This was confirmed in the study of Msanne *et al.* (2015), which demonstrated that *A. thaliana gcs-1* null mutants had a higher GIPC content compared with the wild type. In *P. patens*, GIPCs with different head groups were analysed. Series A GIPCs include species with one hexose (Hex) moiety [Hex or *N*-acetylhexosamine (HexNAc)] that is connected to glucuronic acid (GlcA)-linked IPC, that is, Hex(NAc)-GlcA-IPCs. Series B GIPCs include species with two Hex moieties, of which one may be HexNAc, that is, Hex-Hex(NAc)-GlcA-IPCs. Changes in GIPC profiles were most prominent in the Hex-HexNAc-GlcA-IPC profile (Fig. 4, Supplementary Fig. S4). The wild-type Hex-HexNAc-GlcA-IPC profile consisted mainly of species with a t18:0 LCB in their backbone (Fig. 4A). d18:0, d18:1, and d18:2 LCBs were present in only low amounts in the wild type. The most abundant fatty acids in the wild type were h24:0 (53%), h24:1 (17%), h22:0 (9%), and h20:0 (5%). Other less abundant fatty acids had acyl chain lengths ranging from C20 to C26 that were mostly α-hydroxylated. In the *sd4d-1* Hex-HexNAc-GlcA-IPC profile, molecular species containing a d18:1 or d18:2 LCB were missing (Fig. 4B). Otherwise, the *sd4d-1* profile looked similar to the wild-type profile. *gcs-1* mutants had a comparable Hex-HexNAc-GlcA-IPC profile to that of the wild type (Fig. 4C). However, minimal amounts of species with d18:2/h20:0 (1.5%) ceramide composition emerged in the mutant. Similar changes were observed for GIPC classes with a different head group composition (Supplementary Fig. S4). To summarize, GIPC profiles were affected to a minor degree, which reflected the changes to the ceramide profiles in these mutants.

### *sd4d-1* and *gcs-1* mutants have altered sphingolipid contents

**Fig. 5.**
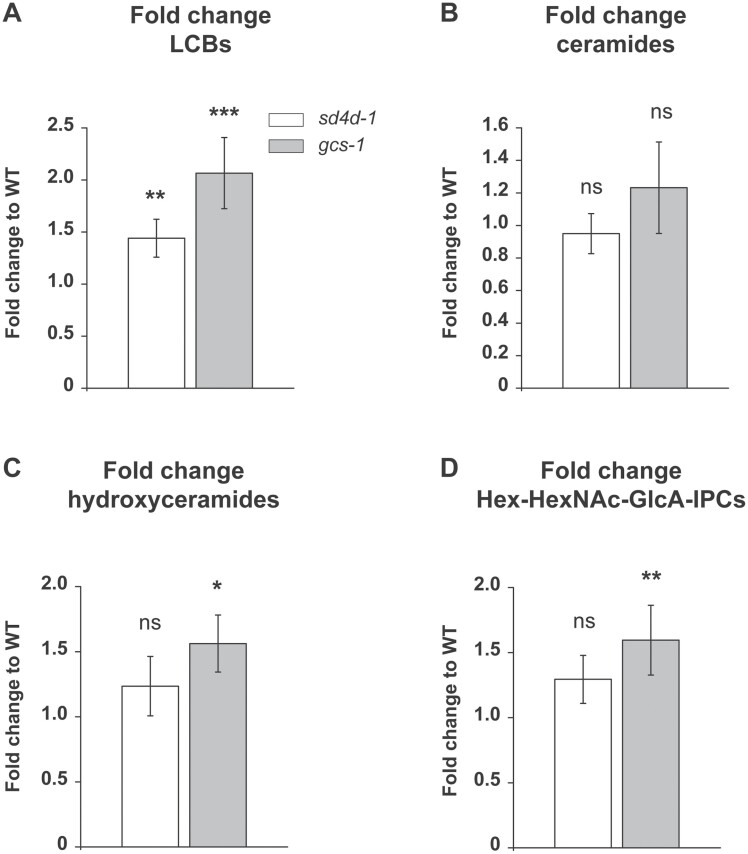
Total contents of LCBs, ceramides, and GIPCs in *P. patens sd4d-1* and *gcs-1*. LCBs, ceramides, hydroxyceramides, and GIPCs were extracted from protonema of 10-day-old wild type (WT), *sd4d-1*, and *gcs-1 P. patens* and analysed with UPLC-nanoESI-MS/MS. Fold changes of (A) LCBs, (B) ceramides, (C) hydroxyceramides, and (D) Hex-HexNAc-GlcA-IPCs relative to the WT were calculated using absolute peak areas. Fold changes are depicted on a linear scale. The WT, which is not shown, was set to a value of 1. Sphingolipid data represent the mean ±SD of measurements from four independent cultivations, each containing protonema material from eight cultivation plates. Statistical analysis was done using a two-tailed Student’s *t*-test. Asterisks indicate significant differences compared with the WT: ***P<0.001, **P<0.01, *P<0.05; ns, not significant (*P*>0.05).

The loss of GlcCers and of sphingolipids with Δ4-unsaturated LCBs influenced the relative profiles of other sphingolipid classes in *gcs-1* and *sd4d-1* mutants (Figs 3, 4). To investigate whether the total sphingolipid contents were also affected, fold changes compared with the wild type were calculated for the individual sphingolipid classes using the absolute peak areas of the analysed compounds (Fig. 5). In *sd4d-1* only LCBs were significantly increased compared with the wild type (Fig. 5A). *sd4d-1* also had a 2-fold higher level of ceramides with d18:0 LCBs and lacked ceramides with d18:1 and d18:2 LCBs (Supplementary Fig. S5A). The total amounts of all other sphingolipid classes were not significantly affected in *sd4d-1* (Fig. 5B–D, Supplementary Fig. S6). In contrast, *gcs-1* showed significant accumulation of LCBs, hydroxyceramides, and Hex-HexNAc-GlcA-IPCs compared with the wild type (Fig. 5A, C, D). *gcs-1* also had a 25-fold higher level of ceramides with a d18:2 LCB relative to the wild type (Supplementary Fig. S5B). Total levels of ceramides, LCB-Ps, Hex-GlcA-IPCs, HexNAc-GlcA-IPCs, and Hex-Hex-GlcA-IPCs were not significantly affected in *gcs-1* compared with the wild type (Fig. 5B, Supplementary Fig. S6). These findings indicated that disruption of PpGCS function influences the total contents of the *P. patens* sphingolipidome more strongly than disruption of PpSD4D.

### *gcs-1* has a more severely impaired growth and development phenotype than *sd4d-1*

The *A. thaliana* LCB Δ4-desaturase mutant does not show a development phenotype (Michaelson *et al.*, 2009). Although mutant floral tissue had reduced GlcCer levels, the knockout did not show physiological defects, leading the authors to conclude that sphingolipids with Δ4-unsaturated LCBs do not have an essential role in *A. thaliana* (Michaelson *et al.*, 2009). Disruption of *A. thaliana* GCS, however, caused seedling lethality and impaired cell differentiation and organogenesis (Msanne *et al.*, 2015). In *A. thaliana*, most GlcCer species have either t18:1 or d18:1 LCBs (Markham *et al.*, 2006). Given that in *P. patens* the d18:2 LCB is the most abundant LCB in GlcCers and that this sphingolipid class was found throughout the plant, PpSD4D and PpGCS were both expected to have major and similar physiological roles in the moss. The sphingolipid data from this study showed that both independent knockout mutants, *sd4d-1* and *gcs-1*, were almost devoid of GlcCers (Fig. 2D).

**Fig. 6.**
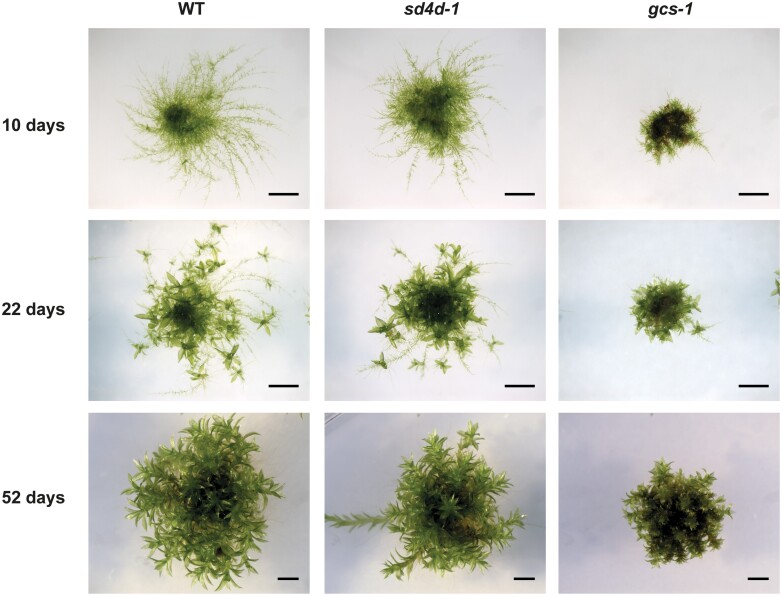
Phenotypes of *P. patens* wild type (WT), *sd4d-1*, and *gcs-1* gametophore and protonema growth. Colonies were grown for 7 weeks and photographed at the indicated time points. Scale bars=2 mm.

To perform phenotype investigations in *P. patens*, colonies were started by placing protonema spot inocula of similar size (~1 mm in diameter) on to BCD medium. After 10 days of growth, wild-type colonies developed long stretched and branched protonema filaments (Fig. 6). After 22 days, the emergence of gametophores was observed. After 52 days, wild-type colonies consisted of fully expanded gametophores that overgrew the protonema. *sd4d-1* showed similar protonema and gametophore development to that of the wild-type control plants (Fig. 6). However, *sd4d-1* protonema filaments appeared shorter and, in consequence, *sd4d-1* colony spread was more restricted than that of the wild type. *gcs-1* mutants had much shorter protonema filaments and had dwarfed gametophores compared with the wild-type and *sd4d-1* plants (Fig. 6).

To quantify the growth of the *P. patens* wild type, *sd4d-1*, and *gcs-1*, protonema was cultivated for 10 days on cellophane-covered BCD plates. All cultivation plates were inoculated with the same amount of starting material. After harvesting, the fresh weight was determined. *sd4d-1* and *gcs-1* mutants generated significantly less biomass than the wild type (Supplementary Fig. S7A, Supplementary Table S5). After lyophilization, the dry weight of the material was also determined, revealing a similar but dampened effect (Supplementary Fig. S7B, Supplementary Table S5). This result indicated that both mutant lines had quantifiable growth defects, and that this phenotype is not solely linked to dry biomass accumulation.

### *gcs-1* has impaired protonema cell differentiation

**Fig. 7.**
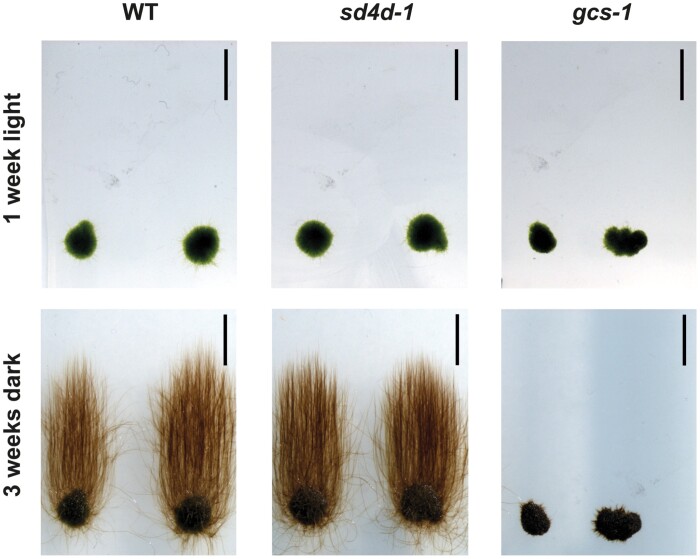
Skotonema development of *P. patens* wild type (WT), *sd4d-1*, and *gcs-1*. Protonema spot inocula (1 mm) of WT, *sd4d-1*, and *gcs-1* lines were placed on BCDAT+2% sucrose and grown under continuous light for 1 week (upper row). Plates were then transferred to the dark and rotated into vertical orientation. Colonies were grown for another 3 weeks to induce skotonema development (lower row). The experiment was repeated three times with similar results. Scale bars=0.5 cm.

The protonema is a two-dimensional filamentous network that consists of two cell types: chloronema cells, which are rich in chloroplasts, and caulonema cells, which have fewer and less developed chloroplasts. The chloronema cells are the initial cells that gradually differentiate into caulonema cells. As mentioned above, *A. thaliana gcs*-*1* plants were impaired in cell differentiation (Msanne *et al.*, 2015). To asses whether cell differentiation was also affected in *P. patens sd4d-1* and *gcs-1*, a dark growth assay was performed. During this assay, cultivation of a subtype of caulonema cells, specified as skotonema cells, is induced by growing plants in the dark. Protonema spot inocula were placed on BCDAT medium supplemented with 2% sucrose and grown for 1 week under continuous light. The culture plates were subsequently transferred to the dark, rotated into vertical orientation, and cultivation was continued for another 3 weeks. After 1 week under continuous light, the wild type and the *sd4d-1* and *gcs-1* mutants developed into dense green protonema colonies of similar size (Fig. 7, upper row). After 3 more weeks of cultivation in the dark and in vertical orientation, the wild type developed long, brown, and unbranched filaments that reached upwards (Fig. 7, lower row). *sd4d-1* colonies looked similar to the wild type, although the mutant filaments seemed to be slightly shorter than the wild-type filaments. In contrast to the wild-type and *sd4d-1* colonies, *gcs-1* colonies failed to develop skotonema filaments. Protonema differentiation ability therefore appeared to be strongly impaired in *gcs-1* mutants but not in *sd4d-1* mutants.

### *sd4d-1* may be impaired in cell elongation

**Fig. 8.**
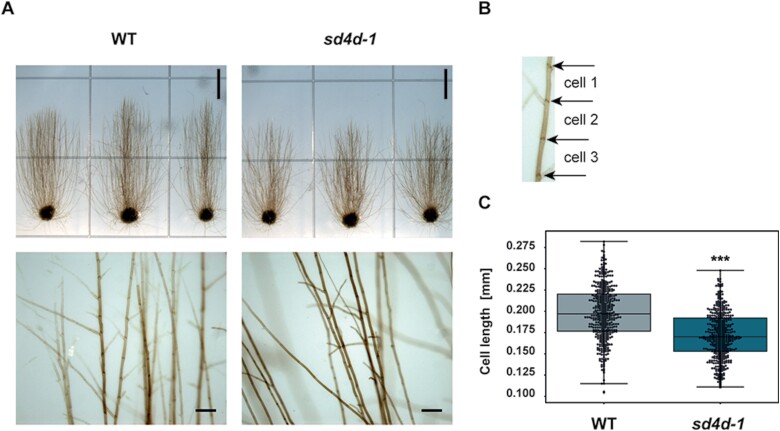
Determination of skotonema cell length of *P. patens* wild type (WT) and *sd4d-1* plants. WT and *sd4d-1* protonema spot inocula were placed on BCDAT+2% sucrose and grown under continuous light for 1 week. Plates were then transferred to the dark and rotated into vertical orientation. (A) Colonies were grown for another 3 weeks to induce skotonema development. Photographs were taken at different magnifications. Scale bars=0.5 cm (upper row) and 0.2 mm (lower row). (B) Skotonema cells are separated by cross-walls. (C) Skotonema cell length measurements of *P. patens* WT and *sd4d-1* plants. Measurements were performed on 428 cells for each line. The experiment was repeated twice with similar results. Statistical analysis was done using a two-tailed Student’s *t*-test. Asterisks indicate significant differences compared with the WT: ***P<0.001.

The *sd4d-1* mutants had slightly shorter skotonema filaments than the wild type (Fig. 7). A possible explanation for the shortened filaments might be that *sd4d-1* cells were generally shorter. To determine the cell lengths, the dark growth experiment was repeated with smaller spot inocula to obtain fewer filaments (Fig. 8A, upper row). This facilitated the examination of individual filaments. The experiment was repeated with the same conditions as described above. Photographs of the filaments were additionally taken at higher magnification to enable the identification of individual cells (Fig. 8A, lower row) and the separating cross-walls (Fig. 8B). A total of 428 cells were measured for each plant line. The mean *sd4d-1* cell length (0.17 mm) was significantly (*P*<0.001) shorter than the mean wild-type cell length (0.2 mm) (Fig. 8C, Supplementary Table S6).

## Discussion

Sphingolipid metabolism has diversified across different plant lineages. An example is the LCB composition of GlcCers, which differs between plant species and tissue types. The causes and consequences of this divergent evolution are still unknown. In bryophytes and Solanaceae, GlcCers are characterized by a Δ4,8-diunsaturated LCB that, by contrast, in *A. thaliana* is present only in sphingolipids of floral tissue (Sperling *et al.*, 2005; Markham *et al.*, 2006; Michaelson *et al.*, 2009; Resemann *et al.*, 2021). In *A. thaliana*, sphingolipids with Δ8-unsaturated LCBs predominate in all sphingolipid classes. Physiologically, LCB Δ8-desaturation has been associated with the response to cold stress and aluminium tolerance (Chen *et al.*, 2012; Sato *et al.*, 2019). However, the role of Δ4-unsaturated LCBs in plants is still elusive. The characterized *A. thaliana* LCB Δ4-desaturase has a restricted expression pattern and the knockout mutant did not reveal a physiologically relevant function in the investigated tissues and conditions (Michaelson *et al.*, 2009). In contrast to *A. thaliana*, *P. patens* GlcCers have high levels of Δ4,8-diunsaturated LCBs (Fig. 1). It was therefore expected that LCB Δ4-desaturase activity would be physiologically more relevant in *P. patens*. Investigation of the *P. patens* loss-of-function mutants for LCB Δ4-desaturase and GCS might therefore give new insights into the metabolic and physiological roles of Δ4-unsaturated molecular species and GlcCers in plants. Both mutant lines lacked nearly all GlcCers but exhibited unexpectedly different phenotypes. Although both enzymes are in the same metabolic pathway, their physiological impact appears to differ greatly.

A recently conducted analysis of the *P. patens* lipidome included GlcCer composition (Resemann *et al.*, 2021). Over 94% of the GlcCer pool consisted of a single molecular species with a Δ4,8-diunsaturated LCB connected to a h20:0 fatty acid moiety, d18:2/h20:0 (Resemann *et al.*, 2021). This finding indicated that GlcCers should be strongly affected by the loss of either PpSD4D or PpGCS. UPLC-nanoESI-MS/MS analyses revealed that GlcCers were nearly absent in both *sd4d* and *gcs* knockout mutant lines (Fig. 2). The results were consistent with findings from the corresponding *A. thaliana* knockouts. The *A. thaliana* LCB Δ4-desaturase mutant had a significant reduction in GlcCer levels in pollen (Michaelson *et al.*, 2009), and *A. thaliana gcs* plants were devoid of all GlcCers (Msanne *et al.*, 2015).

Michaelson *et al.* (2009) speculated that LCB Δ4-desaturation may have a significant role in channelling ceramide substrates into GlcCers in some plants and fungi. Indeed, the observed metabolic changes in the *P. patens sd4d-1* and *gcs-1* mutants were similar to changes in the sphingolipid profiles of the corresponding *Pichia pastoris* knockout mutants (Ternes *et al.*, 2011). The *P. pastoris* LCB Δ4-desaturase knockout, *Δ4Δ*, and the GCS knockout, *gcsΔ*, were also both devoid of GlcCers. This observation confirmed the channelling function of LCB Δ4-desaturation for the yeast *P. pastoris*, whereas our study confirmed the channelling function for the non-vascular plant *P. patens* (Ternes *et al.*, 2011).

The *P. patens sd4d-1* plants were devoid of all unsaturated LCBs (Fig. 3). This observation implied that LCB double-bond insertion in *P. patens* follows a sequential order. The Δ4 double bond appears to be inserted first, followed by insertion of the Δ8 double bond. The d18:1^Δ4^ LCB might therefore act as substrate for the LCB Δ8-desaturase. Inhibition of Δ4 double-bond insertion therefore caused a loss of all LCB double bonds in *sd4d-1* plants. This suggestion is supported by the sphingolipid screen conducted by Islam *et al.* (2012), who found that in *P. patens* d18:1 LCBs, the double bond is present in the Δ4 position. Current data is insufficient to conclude whether the *P. patens* LCB Δ4-desaturase prefers free LCBs or LCBs bound in ceramides as substrates.

Interestingly, although in *sd4d-1* plants GlcCer formation was drastically reduced, residual amounts were still present (Fig. 2). These leftover GlcCers contained d18:0 LCBs (Supplementary Table S2). GlcCers with a d18:0 LCB were not affected by loss of the LCB Δ4-desaturase activity. However, in wild-type and *sd4d-1* plants these molecular species were present only in trace amounts. Surprisingly, the main d18:2/h20:0 GlcCer species was also detected in trace amounts in the *sd4d-1* mutant (Supplementary Table S2). This might be explained by the close functional relation of the LCB Δ4-desaturase to the LCB C-4 hydroxylase. Both enzymes have three characteristic histidine boxes in their active site and are part of a bifunctional enzyme complex in mammals (Ternes *et al.*, 2002). The LCB C-4 hydroxylase might therefore have a low-level desaturase activity that is normally negligible in comparison to that of the LCB Δ4-desaturase but that results in the formation of trace amounts of d18:2/h20:0 GlcCers in *sd4d-1*.

The loss of almost all GlcCers in *sd4d-1* plants indicated that PpGCS preferentially uses ceramides with a Δ4,8-diunsaturated LCB as substrates (Fig. 2, Supplementary Table S2). Furthermore, the accumulation of the d18:2/h20:0 ceramide species in *gcs-1* mutants identified this compound as a putative substrate of PpGCS (Fig. 3). *gcs-1* plants also accumulated d18:2 LCBs and d18:2 LCB-Ps (Fig. 3). These sphingolipid compounds are upstream of the d18:2/h20:0 ceramide species; this result indicates that ceramide formation is a limiting step in complex sphingolipid biosynthesis. The changes in the amount and composition of LCBs and LCB-Ps were relatively minor in both *gcs-1* and *sd4d-1* mutants (Figs 3–5). Therefore, despite the recognized signalling and regulatory functions of LCBs and LCB-Ps, other changes in the lipid profiles of the *gcs* mutant in particular are expected to be responsible for its growth phenotype. Future studies that focus on LCB kinase and phosphatase activity might give more information about the signalling function of LCBs and LCB-Ps in *P. patens*.

Changes in the sphingolipid profiles also affected the morphology of *gcs-1* and *sd4d-1*. Since the *sd4d-1* and *gcs-1* mutants both had significantly reduced GlcCers, it was expected that they would exhibit similar morphological phenotypes. However, surprisingly, while the growth and development of *sd4d-1* were similar to the wild type, *gcs-1* plants had dwarfed gametophores and impaired protonema cell differentiation (Figs 6, 7). In *A. thaliana*, *gcs* RNAi suppression lines with as little as 2% of wild-type GlcCer levels were fertile, whereas *gcs* null mutants were seedling lethal (Msanne *et al.*, 2015). As mentioned earlier, *P. patens sd4d-1* plants had residual GlcCer levels. Cumulative findings from vascular and non-vascular plants suggest that plant performance and cell differentiation are not highly sensitive to the quantity of GlcCers, but a threshold level of GlcCers might be required for proper growth and development (Melser *et al.*, 2011; König *et al.*, 2012; Msanne *et al.*, 2015). Alternatively, the morphological differences observed here may be explained by the stronger accumulation of precursor hydroxyceramides in *gcs-1* compared with *sd4d-1*. It is possible that these precursors are cytotoxic when present in high levels and may therefore inhibit plant growth.

In summary, our findings show that LCB Δ4-desaturation is an important regulatory mechanism in *P. patens* to channel ceramides into GlcCer formation. Although *P. patens sd4d-1* and *gcs-1* plants were both mostly devoid of GlcCers, the two mutant lines had substantially different phenotypes; further work is needed to establish whether these differences are due to substrate accumulation or a threshold level of GlcCer product. Additionally, despite the fact that GlcCers with a Δ4,8-diunsaturated LCB are abundant membrane compounds in *P. patens*, their complete abolishment did not interfere with plant survival. This puts into question their quantitative relevance in plants. *Physcomitrium patens*, with its simple morphology and clearly distinguishable complex sphingolipid composition, represents a valuable model organism for future study and understanding of the diversification of plant sphingolipid metabolism.

## Supplementary data

The following supplementary data are available at *JXB* online.

Fig. S1. Prediction data for transmembrane domains and gene expression.

Fig. S2. *sd4d* and *gcs* mutant characterization.

Fig. S3. GlcCer content of *P. patens* wild type and *sd4d* and *gcs* mutants.

Fig. S4. GIPC profiles of *P. patens* wild type, *sd4d-1*, and *gcs-1*.

Fig. S5. Total content of LCBs in *P. patens sd4d-1* and *gcs-1* ceramides.

Fig. S6. Total contents of LCB-Ps and other GIPC classes in *sd4d-1* and *gcs-1*.

Fig. S7. Fresh weight and dry weight protonema biomass of *P. patens* wild type, *sd4d-1*, and *gcs-1*.

Table S1. Complete list of all multiple reaction monitorings measured for sphingolipid analysis.

Table S2. Raw data (absolute) from sphingolipid analysis in *P. patens*.

Table S3. Raw data (relative) from sphingolipid analysis in *P. patens*.

Table S4. Raw data (absolute) from GlcCer analysis in *P. patens sd4d* and *gcs* mutants.

Table S5. Raw data from biomass determination in *P. patens*.

Table S6. Raw data from skotonema cell length measurement in *P. patens*.

erab238_suppl_Supplementary_Data

erab238_suppl_Supplementary_Figures

## Data Availability

All data supporting the findings of this study are available within the paper and within its supplementary material available online. Mutant material is available from the corresponding author, Ivo Feussner, upon request.

## Abbreviations

GCS: glycosylceramide synthase
GlcA: glucuronic acid
GlcCer: glycosylceramide
GIPC: glycosyl inositolphosphorylceramide
Hex: hexose
HexNAc: *N*-acetylhexosamine
IPC: inositolphosphorylceramide
LCB: long-chain base
LCB-P: long-chain base phosphate
MS/MS: triple quadrupole tandem mass spectrometry
nanoESI: nanoelectrospray ionization
UPLC: ultra-performance liquid chromatography
UTR: untranslated region
