# Supplementary material for: Sphingolipid Δ4-desaturation is an important metabolic step for glycosylceramide formation in Physcomitrium patens
Source: J Exp Bot. 2021 Jun 10;72(15):5569–83. doi: 10.1093/jxb/erab238 (PMC8318264; doi:10.1093/jxb/erab238)
Supplement: erab238_suppl_Supplementary_Figures [file erab238_suppl_supplementary_figures.pdf]

Supplementary data

Supplementary figures

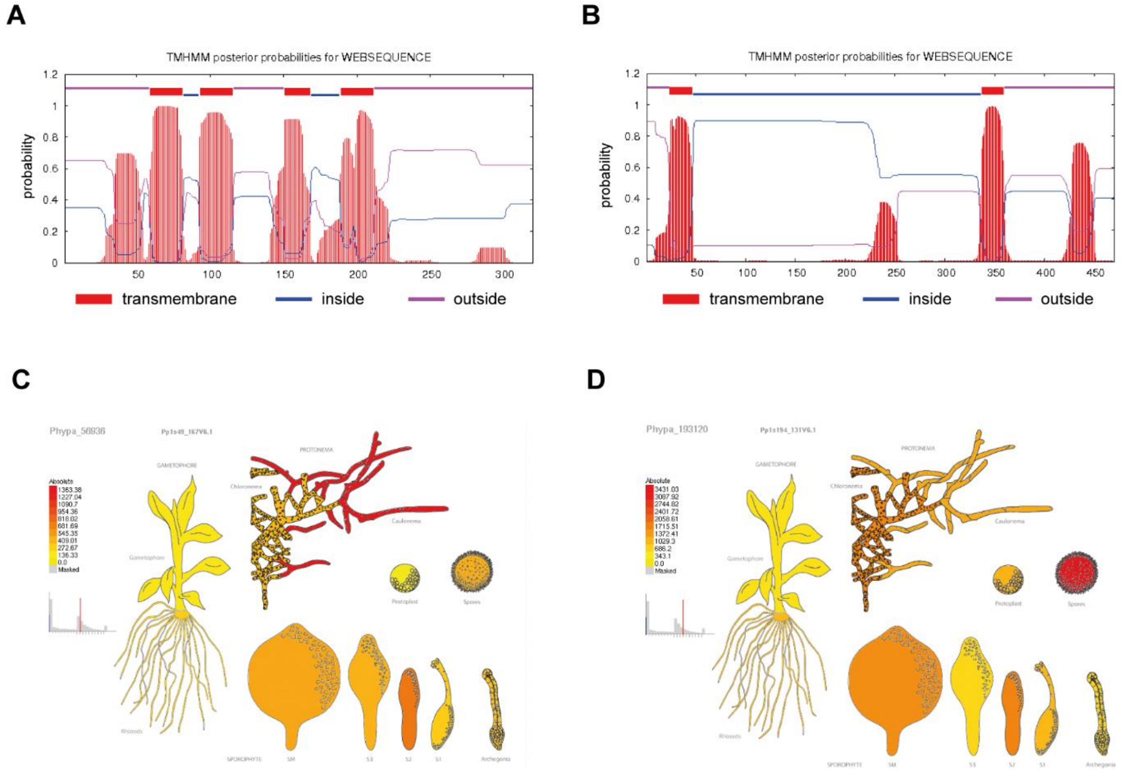

Fig. S1. Prediction data for transmembrane domains and gene expression. Transmembrane domain prediction was done for (A) *Pp*SD4D and (B) *Pp*GCS using TMHMM webtool. Gene expression for (C) *PpSD4D* and (D) *PpGCS* in *P. patens* organs was predicted using eFP browser.

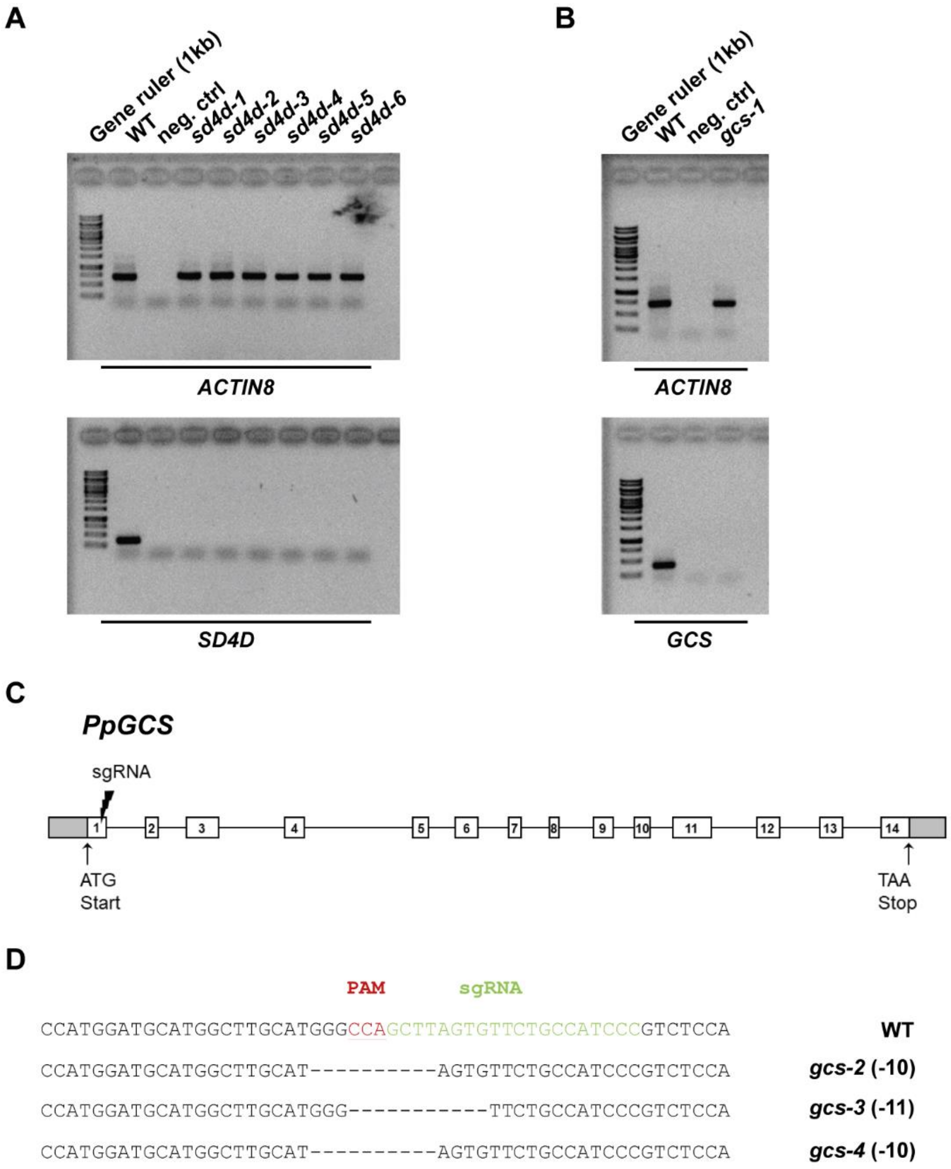

Fig. S2. *sd4d* and *gcs* mutant characterisation. Complete gel picture of (A) *PpSD4D* and (B) *PpGCS* transcript determination by real-time PCR. *ACTIN8* was used as reference gene and water as negative control (neg. ctrl). (C) CRISPR-Cas9 gene editing strategy for *PpGCS* targeting. Single-guide RNA (sgRNA) was designed to target the first exon of *PpGCS*. White boxes indicate exons, grey boxes indicate untranslated regions and lines indicate introns. (D) Sequencing of the targeted locus revealed three *gcs* mutants with frame shift deletions.

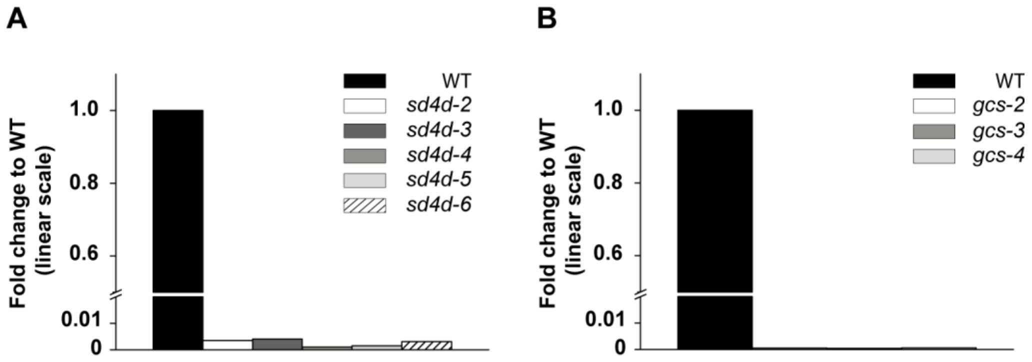

Fig. S3. GlcCer content of *P. patens* wild type, *sd4d,* and *gcs* mutants. Glycosylceramides (GlcCers) were extracted from protonema of ten-day-old wild type (WT), *sd4d-2, -3, -4, -5, -6* and *gcs-2, -3, -4 P. patens* and analysed with UPLC-nanoESI-MS/MS. Fold changes of (A) *sd4d* GlcCers and (B) *gcs* GlcCers to WT GlcCers were calculated using absolute peak areas. Fold changes are depicted in linear scale. The WT is set to 1. Sphingolipid data represent the measurement from one cultivation.

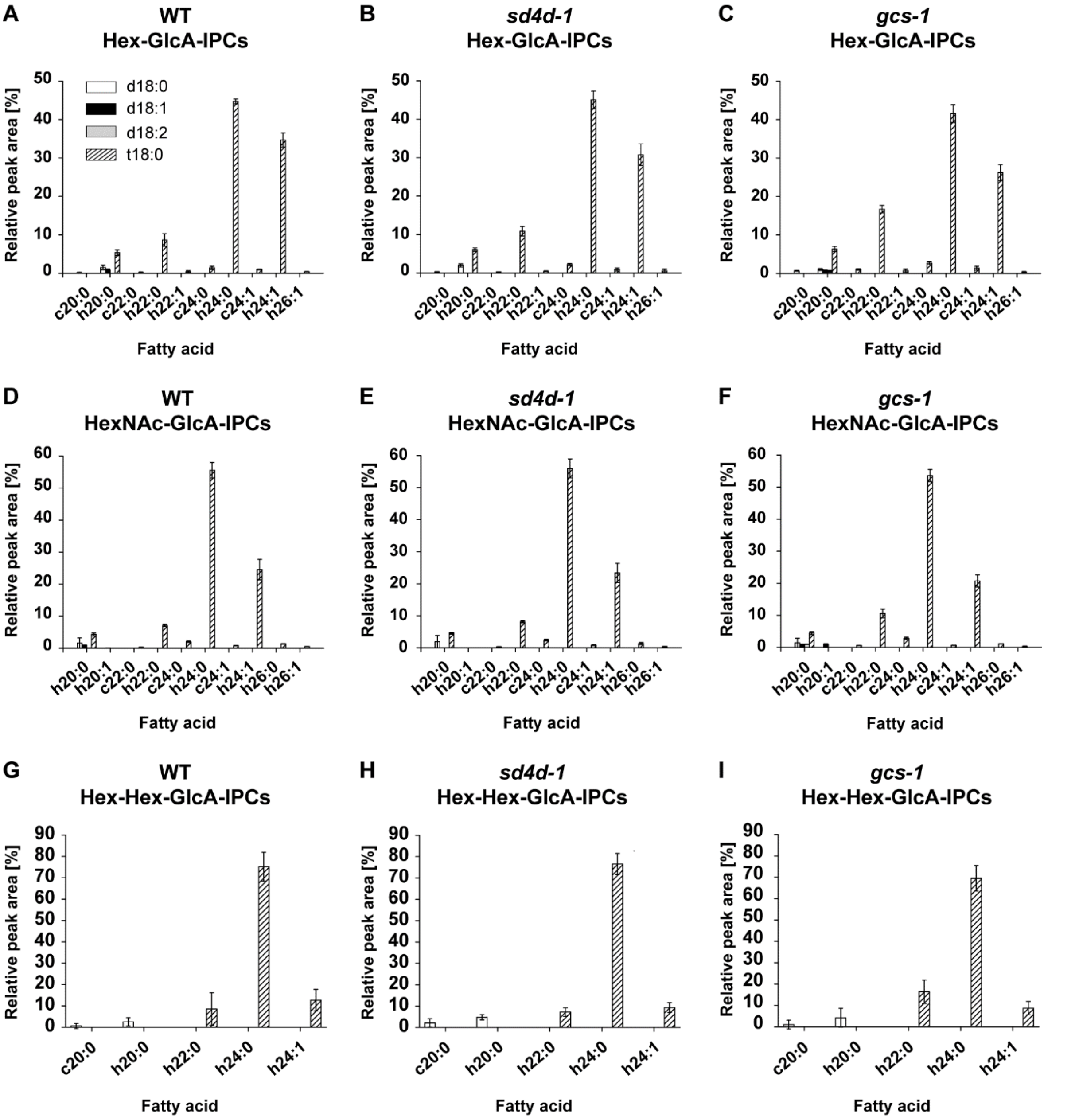

Fig. S4. GIPC profiles of *P. patens* wild type, *sd4d-1*, and *gcs-1*. (A-C) Glycosyl inositolphosphorylceramides (GIPCs) were extracted from protonema of ten-day-old wild type (WT), *sd4d-1*, and *gcs-1 P. patens* and analysed with UPLC-nanoESI-MS/MS. GIPC molecular species are shown with their LCB (column colour) and fatty acid (x-axis) moieties. Dihydroxy LCBs are indicated by a ‘d’ and trihydroxy LCBs are indicated by a ‘t’. Molecular species with unhydroxylated fatty acids are indicated by a ‘c’ and molecular species with α-hydroxylated fatty acids are indicated by an ‘h’. (A-C) Relative Hex-GlcA-IPC profiles of (A) WT, (b) *sd4d-1*, and (C) *gcs-1*. (D-F) Relative HexNAc-GlcA-IPC profiles of (D) WT, (E) *sd4d-1*, and (F) *gcs-1.* (G-I) Relative Hex-Hex-GlcA-IPC profiles of (G) WT, (H) *sd4d-1*, and (I) *gcs-1.* Only molecular species with a peak area ≥ 0.5 % in at least one of the three lines were included in GIPC graphs. Sphingolipid data represent the mean ± SD of measurements from four independent cultivations each containing protonema material from eight cultivation plates. Abbreviations are as follows: GlcA: glucuronic acid; Hex: hexose; HexNAc. *N*-acetylhexosamine, IPCs: inositolphosphorylceramides.

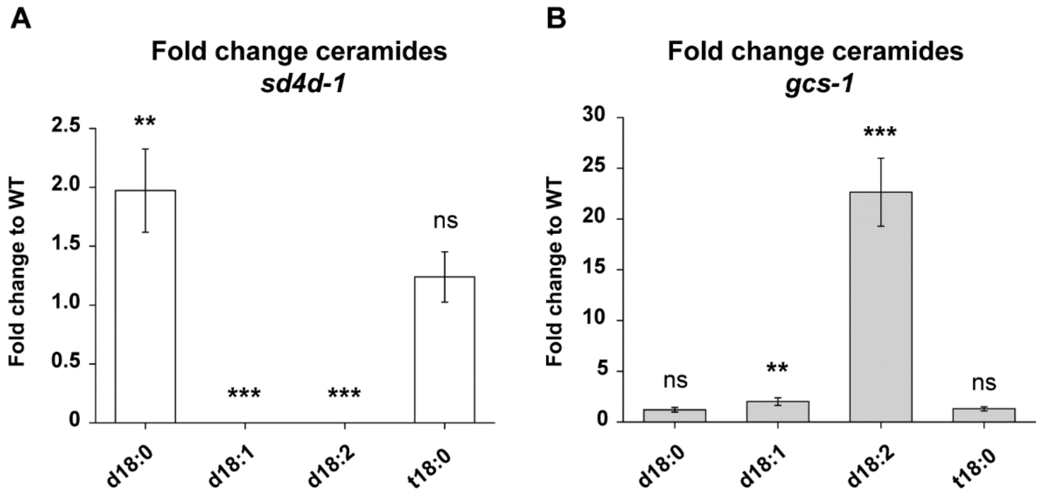

Fig. S5. Total content of LCB moieties in *P. patens sd4d-1* and *gcs-1* ceramides. Ceramides were extracted from protonema of ten-day-old wild type (WT), *sd4d-1*, and *gcs-1 P. patens* and analysed with UPLC-nanoESI-MS/MS. Species with the same LCB moiety were summed up. Fold changes of ceramide LCB moieties from (A) *sd4d-1* and (B) *gcs-1* to the WT were calculated using absolute peak areas. Fold changes are depicted in linear scale. The WT, which is not shown, is set to 1. Sphingolipid data represent the mean ± SD of measurements from four independent cultivations each containing protonema material from eight cultivation plates. Statistical analysis was done using a two-tailed Student's *t*-test. Asterisks indicate different significance levels with *** significance at $P < 0.001$, ** significance at $P < 0.01$, and not significant (ns) at $P > 0.05$ compared to the WT.

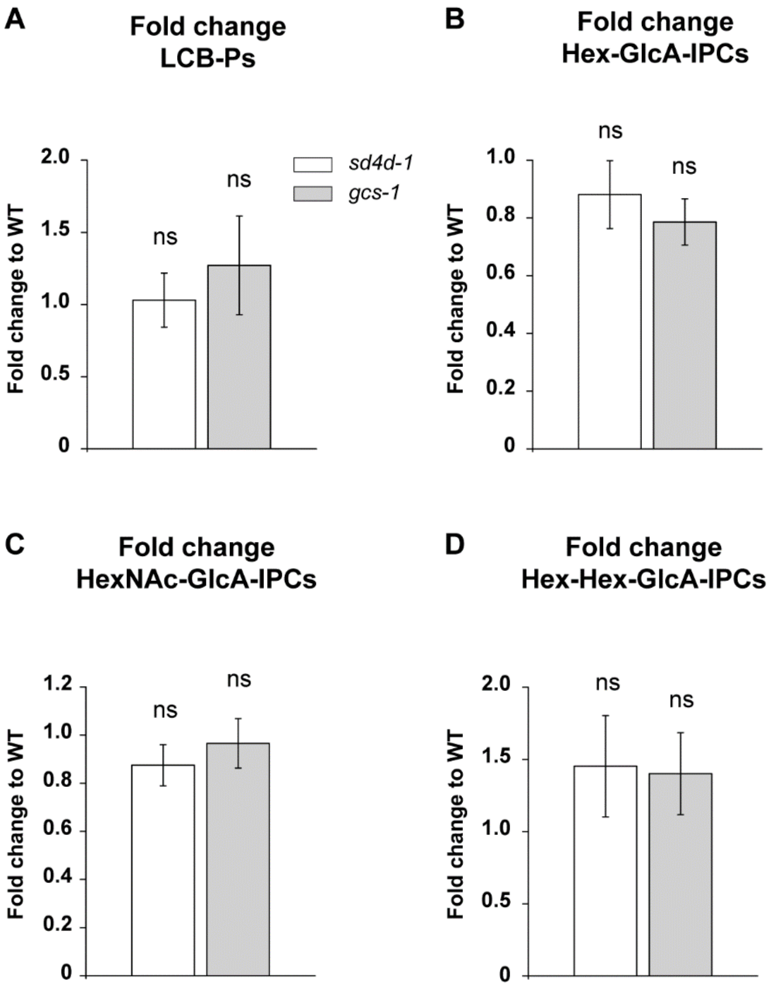

Fig. S6. Total contents of LCB-Ps and other GIPC classes in *sd4d-1* and *gcs-1*. Phosphorylated long-chain bases (LCB-Ps), and glycosyl inositolphosphorylceramide (GIPCs) were extracted from protonema of ten-day-old wild type (WT), *sd4d-1*, and *gcs-1 P. patens* and analysed with UPLC-nanoESI-MS/MS. Fold changes of (A) LCB-Ps, (B) Hex-GlcA-IPCs, (C) HexNAc-GlcA-IPCs, and (D) Hex-Hex-GlcA-IPCs to the WT were calculated using absolute peak areas. Fold changes are depicted in linear scale. The WT, which is not shown, is set to 1. Sphingolipid data represent the mean ± SD of measurements from four independent cultivations each containing protonema material from eight cultivation plates. Statistical analysis was done using a two-tailed Student's *t*-test. Letters indicate no significance (ns) to the WT with $P > 0.05$. Abbreviations are as follows: GlcA: glucuronic acid; Hex: hexose, HexNAc: *N*-acetylhexosamine, IPCs: inositolphosphate.

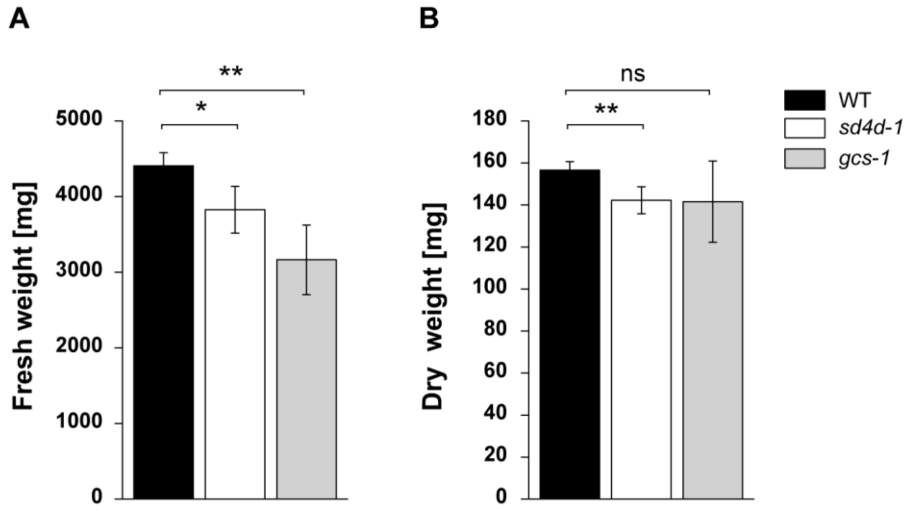

Fig. S7. Fresh weight and dry weight protonema biomass of *P. patens* wild type, *sd4d-1*, and *gcs-1*. Growth capacities of the wild type (WT), *sd4d-1*, and *gcs-1* lines were quantified by collecting ten-day-old protonema grown on cellophane-covered BCD medium and determining (A) the fresh weight. After lyophilising, (B) the protonema dry weight was determined. Data represent the mean ± SD of measurements from four independent cultivations each containing protonema material from eight cultivation plates. Statistical analysis was done using a two-tailed Student's *t*-test. Asterisks indicate different significance levels with ** significance at P < 0.01, * significance at P <0 .05, and not significant (ns) at P > 0.05 compared with the WT.
